# Distinct requirement of Runx complexes for TCRβ enhancer activation at distinct developmental stages

**DOI:** 10.1038/srep41351

**Published:** 2017-02-02

**Authors:** Wooseok Seo, Sawako Muroi, Kaori Akiyama, Ichiro Taniuchi

**Affiliations:** 1 Laboratory for Transcriptional Regulation, RIKEN Center for Integrative Medical Sciences (IMS), 1-7-22 Suehiro-cho, Tsurumi-ku, Yokohama 230-0045, Japan

## Abstract

A TCRβ enhancer, known as the Eβ enhancer, plays a critical role in V(D)J recombination and transcription of the *Tcrb* gene. However, the coordinated action of *trans*-acting factors in the activation of Eβ during T cell development remains uncharacterized. Here, we characterized the roles of Runx complexes in the regulation of the Eβ function. A single mutation at one of the two Runx binding motifs within the Eβ severely impaired *Tcrb* activation at the initiation phase in immature thymocytes. However, TCRβ expression level in mature thymocytes that developed under such a single Runx site mutation was similar to that of the control. In contrast, mutations at two Runx motifs eliminated Eβ activity, demonstrating that Runx complex binding is essential to initiate Eβ activation. In cells expressing *Tcrb* harboring rearranged V(D)J structure, Runx complexes are dispensable to maintain TCRβ expression, whereas Eβ itself is continuously required for TCRβ expression. These findings imply that Runx complexes are essential for Eβ activation at the initiation phase, but are not necessary for maintaining Eβ activity at later developmental stages. Collectively, our results indicate that the requirements of *trans*-acting factor for Eβ activity are differentially regulated, depending on the developmental stage and cellular activation status.

Transcriptional control of the spatio-temporal expression of developmentally regulated genes involves dynamic communication between DNA regulatory elements (*cis*-regulatory elements), such as enhancers, and regulatory proteins (*trans*-regulatory elements) including chromatin modifiers and transcriptional factors. Enhancers are classically defined as DNA elements that can activate the transcription of their target loci irrespective of their orientation or distance from the transcriptional start site^1^.

During T and B lymphocyte development, antigen receptor genes such as T cell receptor (*Tcr*) genes and immunoglobulin (*Ig*) genes are assembled from variable (V), diversity (D) and joining (J) gene segments by a process referred to as V(D)J recombination^2^,^3^, which is regulated in a highly ordered manner^4^. During T cell development, rearrangement of *Tcrb* locus occurs first in CD4^−^CD8^−^ double-negative (DN) thymocytes, and then *Tcra* rearrangement follows during transition into CD4^+^CD8^+^ double-positive (DP) thymocytes only after the functional assembly of a *Tcrb* allele is secured by a process known as β selection. At the *Tcrb* locus, the expression of functionally assembled *Tcrb* alleles prevents further V to DJ recombination on the second allele to ensure the mono-specificity of the antigen receptor (a process known as allelic exclusion)^5^. Thus, similar to other developmentally regulated genes, a highly ordered V(D)J assembly might be controlled by combinational regulation of *cis*-regulatory elements and trans-acting factors.

Within the ~670 kb *Tcrb* locus, there are twenty-one individual Vβ gene segments, which spread over the ~300 kb of the 5′ side of the locus and contain their own promoters, and duplicated Dβ-Jβ-Cβ regions within ~26 kb of the 3′ end of the locus. A single enhancer element, the TCRβ enhancer (Eβ), located at the 3′ side of the Cβ2 region, has been shown to play an essential role in the recombination and transcription of DβJβ clusters^6^,^7^, while a promoter that neighbors the Dβ1 gene segment has been shown to govern these two reactions at the Dβ1 region but not at the Dβ2 region^8^. Thus Eβ activates either the Dβ1 or Dβ2 promoter to initiate recombination and transcription at each DβJβ cluster. In contrast, the deletion of Eβ has no measurable effect on germline transcription at upstream Vβ gene segments in T cell progenitors harboring the germline structure of the *Tcrb* allele^9^. However, another study using a bacterial artificial chromosome (BAC) transgene with a rearranged V(D)J region indicated that Eβ is required to activate Vβ promoters at later stages of thymocyte development^10^. Thus functional interaction between Eβ and Vβ promoters may be differentially regulated according to genomic structures or developmental stages. To understand the molecular mechanism that governs the activation of the Dβ and Vβ promoters by the enhancer Eβ, it is critical to investigate the function of *trans*-acting factors that bind to the enhancer.

Ets-1 and Runx transcriptional factors have been implicated to function as *trans*-factors for Eβ. Mutagenesis studies with reporter transfection assays and transgenic substrate have demonstrated that both Ets-1 and Runx binding motifs are essential for Eβ enhancer activity^11^,^12^. In an early study, we employed conditional knockout strategies in mice and showed that the inactivation of *Runx1* in DN thymocytes by the *Lck*-*cre* transgene resulted in a decrease of DN4 thymocytes, while DN3 cell numbers were not affected^13^, indicating that Runx1 is required for the proliferation of thymocytes at the DN3–DN4 transition.

In this study, we report that Eβ-mediated TCRβ locus activation in T cell progenitors requires Runx binding sites, but the Eβ enhancer becomes independent of Runx complexes to maintain TCRβ expression in mature T cells. Thus, the functional requirements of Runx complexes for Eβ activation are distinct at different stages of the *Tcrb* locus, illustrating distinct regulation of Eβ activity at the initiation versus maintenance phases by *trans*-acting factors.

## Results

### Runx binding is necessary for Eβ activation to initiate TCRβ expression

**Figure 1:**
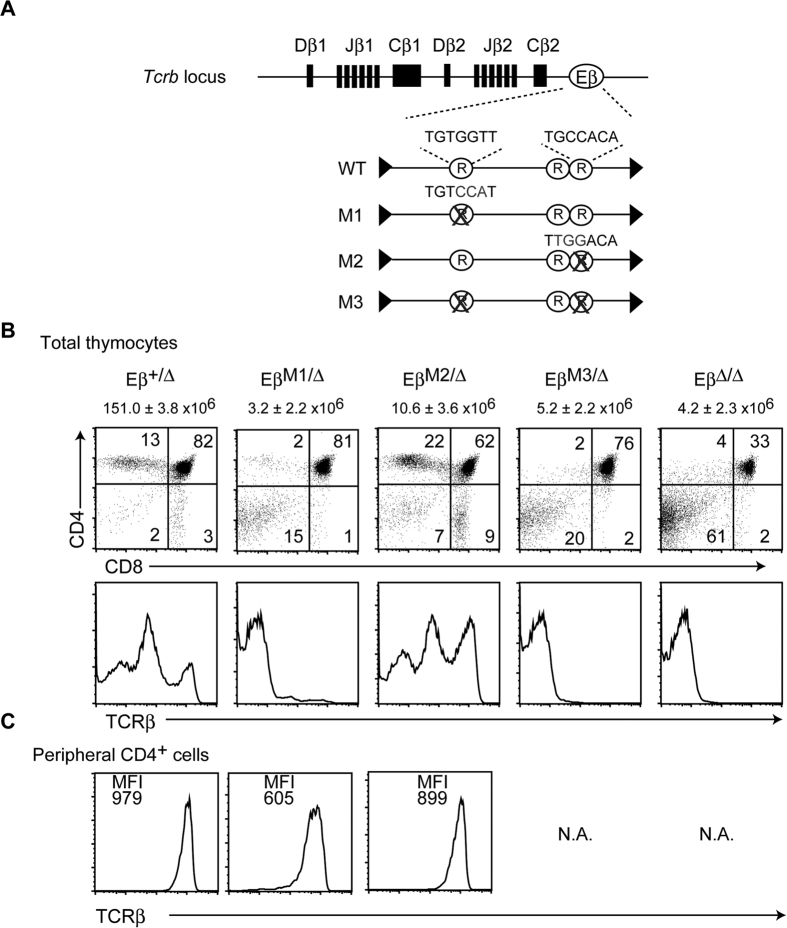
Importance of Runx recognition sites for Eβ enhancer activation. (**A**) Schematic map of the *Dβ, Jβ* and *Eβ* regions at the mouse *Tcrb* locus (top line). The lower magnified lines represent the ~600 bp Eβ enhancer region. Three putative Runx binding motifs are indicated as circles marked as R. Replaced nucleotide sequence at the Runx sites in the *Eβ*^*M1*^ and *Eβ*^*M2*^ mutations are shown above. The *Eβ*^*M1*^ and *Eβ*^*M2*^ mutations were combined in the *Eβ*^*M3*^ mutation. The filled triangle represents loxP sequences. (**B**) Total thymocytes from *Eβ*^+/*Δ*^, *Eβ*^*M1*/*Δ*^, *Eβ*^*M2*/*Δ*^, *Eβ*^*M3*/*Δ*^ and *Eβ*^*Δ*/*Δ*^ mice were stained for surface CD4, CD8 and TCRβ. The representative CD4 and CD8 expression profiles of each mouse are shown as dot blot with the average number of total thymocytes. Surface TCRβ expression on total thymocytes is shown in histograms. (**C**) The expression of the surface αβTCR complex on CD4^+^ lymph node cells was analyzed. N.A.: not available.

Accumulation of DN3 cells expressing a lower percentage of intracellular TCRβ chain (i.c.TCRβ) in the thymus of *Runx1*^*f*/*f*^:*Lck*-*cre* mice^13^ suggested that Runx1 is involved in the initiation of *Tcrb* activation. In contrast, an equivalent level of surface TCRβ on cells lacking Cbfβ protein^14^, the essential binding partner of all Runx proteins^15^, indicated that the function of Runx complexes is dispensable for TCRβ expression in mature T cells. Such distinct roles of Runx complexes for *Tcrb* expression at distinct stages prompted us to examine the roles of Runx complexes to control Eβ function, as well roles of Eβ in *Tcrb* expression during T cell development, particularly at later developmental stages. We therefore first addressed Runx sites (5′-PuACCACG/A-3′) within the Eβ for their requirement for enhancer function by targeting mutations by homologous recombination in mouse embryonic stem (ES) cells (Fig. 1A and Supplemental Figure 1). M1 and M2 mutations were designed to abrogate the core CCAC sequence of Runx binding motifs in βE4 and βE6 elements^16^, respectively (Fig. 1A). In the M3 mutation, the M1 and M2 mutations were combined. At the same time, the 560-bp core Eβ sequences were flanked with loxP sequences for Cre-mediated conditional deletion.

Consistent with previous reports^6^,^7^, the deletion of Eβ (*Eβ*^*Δ/Δ*^) in the germline resulted in a loss of surface TCRβ expression on thymocytes (Fig. 1B). Differentiation of a small number of CD4^+^CD8^+^ DP thymocytes in the *Eβ*^*Δ/Δ*^ mice has been shown to be dependent on the expression of γδTCR complex^17^. Similarly, *Eβ*^*M3*/*Δ*^ mice showed a severe reduction in thymocyte number and a complete loss of surface TCRβ^+^ cells (Fig. 1B). Therefore, no cells expressing αβTCR complexes were detected in the peripheral lymphoid tissues of *Eβ*^*Δ/Δ*^ and *Eβ*^*M3*/*Δ*^ mice. These phenotypes were completely recapitulated in *Eβ*^*M3*/*M3*^ mice (data not shown). In the thymus from the *Eβ*^*M1*/*Δ*^ and *Eβ*^*M2*/*Δ*^ mice, thymocyte numbers were reduced with an increase in the CD4CD8^−^ DN cell proportion, suggesting a partial block at the CD4^−^CD8^−^ DN to CD4^+^CD8^+^ DP transition. In contrast to *Eβ*^*M3*/*Δ*^ mice, a significant number of CD4^+^CD8^+^ DP thymocytes expressing surface αβTCR complex and mature thymocytes were detected in both *Eβ*^*M1*/*Δ*^ and *Eβ*^*M2*/*Δ*^ mice. Furthermore, the levels of surface αβTCR expression on T cells in the peripheral lymphoid tissues from these mice were similar to that from control animals (Fig. 1C).

**Figure 2:**
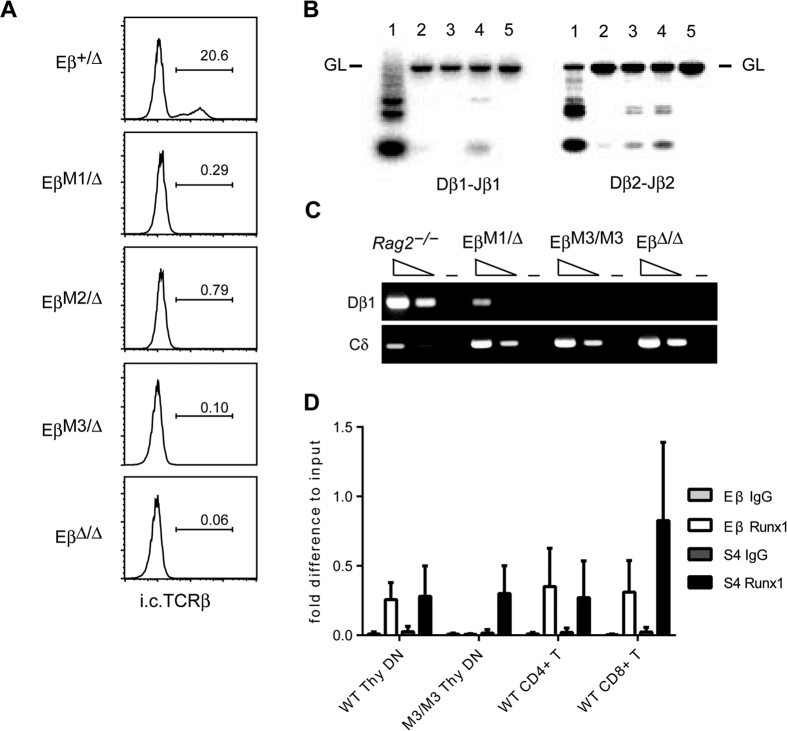
Effect of Runx site mutations on DJ recombination and germline transcription. (**A**) Histograms showing the expression of intracellular TCRβ (i.c.TCRβ) in a CD25^+^ CD44^−^ DN3 subset from mice with the indicated genotype. (**B**) Semi-quantitative DNA-PCR analyses for analyzing Dβ to Jβ rearrangement in CD25^+^ CD44^−^ DN3 subsets from *Eβ*^+/*Δ*^(lane 1), *Eβ*^*Δ*/*Δ*^ (lane 2), *Eβ*^*M1*/*Δ*^ (lane 3), *Eβ*^*M2*/*Δ*^ (lane 4) and *Eβ*^*M3*/*Δ*^ (lane 5) mice. The bar indicates the position corresponding to the germline (GL) configuration. (**C**) The germline transcript of the Dβ1 region in CD4^−^CD8^−^ DN cells isolated from Rag2^−/−^, *Eβ*^*M1*/*Δ*^, *Eβ*^*M3*/*M3*^ and *Eβ*^*Δ*/*Δ*^ mice are shown. The germline transcript of Cδ region was used as control. RNA without reverse-transcriptase reaction is shown in lanes indicated as (−). (**D**) Chromatin immunoprecipitation assay (ChIP) to detect Runx1 binding to Eβ and *Cd4* silencer (*S4*). CD4^−^CD8^−^ DN cells isolated from *wild*-*type* and *Eβ*^*M3*/*M3*^ mice were used to prepare chromatin DNA. Chromatin DNA was immunoprecipitated with the IgG control or anti-Runx1 antibody and was used as the template for qPCR amplification. The *Cd4* silencer region as well as peripheral CD4^+^ and CD8^+^ T cells (WT) were used as controls for Runx1 binding. Combined data from three independent ChIP experiments is shown.

While more than 20% of DN3 cells from control *Eβ*^+/*Δ*^ mice expressed i.c.TCRβ, only 0.29% and 0.79% of DN3 cells from *Eβ*^*M1*/*Δ*^ and *Eβ*^*M2*/*Δ*^ mice expressed i.c.TCRβ, respectively (Fig. 2A). Since a complete lack of Eβ affects recombination and transcription mainly at DβJβ region, we examined impact of *Eβ*^*M1*^ and *Eβ*^*M2*^ mutation on these reactions. In *Eβ*^*M1*/*Δ*^ and *Eβ*^*M2*/*Δ*^ mice, both Dβ1 to Jβ1 and Dβ2 to Jβ2 rearrangements were severely inhibited, albeit to a lesser extent compared to thymocytes harboring the *Eβ*^*M3*^ or *Eb*^*Δ*^ mutation (Fig. 2B). Similarly, germline transcription of the Dβ1 region was partially decreased by *Eβ*^*M1*^ mutation, whereas it was undetectable in *Eβ*^*M3*/*M3*^ and *Eβ*^*Δ*/*Δ*^ thymocytes (Fig. 2C). Furthermore chromatin immunoprecipitation assay (ChIP) with anti-Runx1 antibody showed that only *Cd4* silencer (designated as *S4* in Fig. 2D), a well-characterized *cis*-regulatory region for Runx1 binding in DN thymocytes^18^, was enriched from *Eβ*^*M3*/*M3*^ DN thymocytes, while both the *Eβ* enhancer and the *Cd4* silencer were enriched from control cells (Fig. 2D). Runx1 bindings to these regions were also observed in peripheral CD4^+^ and CD8^+^ T cells. Collectively, these results indicate that Runx binding is essential for the activation of the *Eβ* enhancer and subsequent Dβ to Jβ rearrangement at the *Tcrb* locus, and two Runx sites in the Eβ are partially redundant in their function. It is noteworthy that the loss of one functional Runx binding site did not show significant effects on *Tcrb* expression in mature T cells, while it led to a severe impairment of initial Eβ activation.

### Eβ function during T cell development

**Figure 3:**
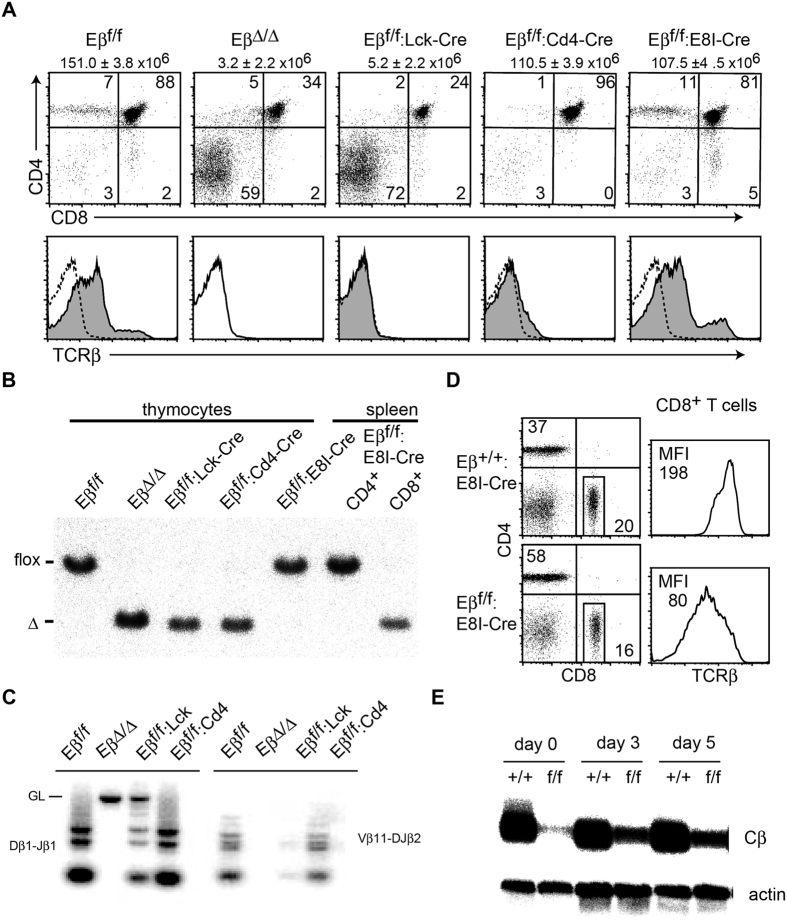
Effect of conditional deletion of Eβ at distinct developmental stages on TCRβ expression. (**A**) Expression levels of CD4, CD8 and TCRβ on total thymocytes from *Eβ *^*f*/*f*^, *Eβ*^*Δ*/*Δ*^, *Eβ *^*f*/*f*^:*Lck*-*Cre, Eβ *^*f*/*f*^:*Cd4*-*Cre* and *Eβ *^*f*/*f*^:*E8I*-*Cre* mice are shown. TCRβ expression in *Eβ*^*Δ*/*Δ*^ mice is shown as a dotted line in the histogram as a control. (**B**) DNA from total thymocytes and sorted CD4^+^ and CD8^+^ splenocytes from mice with the indicated genotype was analyzed for the efficiency of Cre-mediated deletion of Eβ by Southern blot. The bar indicates the position corresponding to the *Eβ *^*f*^ and *Eβ*^*Δ*^ allele. (**C**) DNA-PCR analyses for analyzing Dβ1-Jβ1 and Vβ11-DJβ2 recombination in sorted CD25^+^CD44^−^ DN3 thymocytes from indicated mice. The bar indicates the position corresponding to the germline configuration. (**D**) Expression levels of TCRβ on CD8^+^ lymph node cells from *Eβ *^+/+^:*E8I*-*Cre* and *Eβ *^*f*/*f*^:*E8I*-*Cre* mice are shown as histograms with mean fluorescent intensity (MFI) at the left upper corner. (**E**) Northern blot showing TCRβ transcripts detected by the Cβ probe. CD8^+^ splenocytes from *Eβ *^+/+^:*E8I*-*Cre* and *Eβ *^*f*/*f*^:*E8I*-*Cre* mice were activated by TCR stimulation. Total RNA was prepared before and three or five days after stimulation. Five micrograms of total RNA was loaded in each lane. The *actin* mRNA was used as the loading control.

Minor effects of Runx deficiency as well as of M1 and M2 mutations on *Eβ* function in mature T cells challenge the requirement of Eβ for the maintenance of *Tcrb* expression. To examine Eβ function during T cell development, we used mice harboring *Eβ *^*flox*^ alleles and three Cre transgenic lines, including *E8I*-*Cre*, whose expression is detected specifically in mature CD8-lineage cells after downregulation of CD24/HSA marker (Supplementary Figure 2). Southern blot analyses confirmed that nearly all splenic CD8^+^ T cells of *Eβ *^*f*/*f*^:*E8I*-*Cre* mice underwent Cre-mediated removal of the Eβ element, while conversion to the Eβ^Δ^ allele in total thymocytes was less than 2% due to small fraction of mature CD8-lineage cells (Fig. 3B).

The removal of *Eβ* at the DN stage by *Lck*-*Cre* and at the DP stage by *Cd4*-*Cre* resulted in a loss of surface αβTCR expression on DP thymocytes (Fig. 3A). Contrary to germline *Eβ* deletion, DN3 thymocytes from *Eβ *^*f*/*f*^:*Lck*-*Cre* mice underwent a marked level of Dβ to Jβ rearrangement, although Vβ to DβJβ assembly was severely inhibited (Fig. 3C). The deletion of Eβ in maturing thymocytes by *E8I*-*Cre* resulted in a significant downregulation of surface TCRβ expression (Fig. 3D), consistent with a ten-fold reduction of *Tcrb* mRNA in splenic CD8^+^ T cells lacking Eβ (Fig. 3E). These results confirmed the continuous requirement of Eβ in the maintenance of *Tcrb* expression in mature T cells.

### Eβ-independent reactivation of *TCRβ* gene in activated T cells

**Figure 4:**
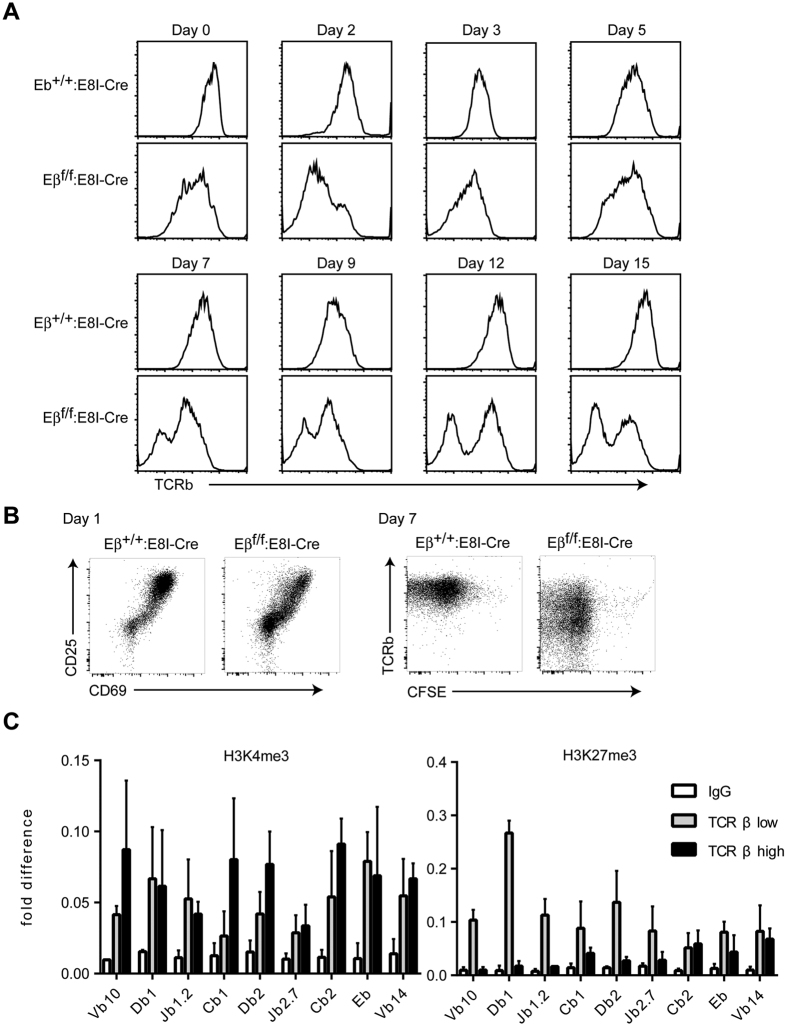
Kinetics of TCRβ expression in the absence of the Eβ after TCR stimulation. (**A**) Splenic CD8^+^ T cells from *Eβ *^+/+^:*E8I*-*Cre* and *Eβ *^*f*/*f*^:*E8I*-*Cre* mice were stimulated with immobilized anti-CD3 and soluble anti-CD28 antibodies. Two days after stimulation, cells were harvested and were kept in culture with medium supplemented with 20 units/ml of mIL-2. TCRβ expression kinetics after stimulation are shown as histograms. (**B**) Dot plots showing CD25 and CD69 activation markers, and TCRb expression and CFSE as a marker for cell proliferation at the indicated days. (**C**) ChIP assay measuring H3K4 and K27 tri-methylation levels at indicated regions in the *Tcrb* locus in TCRβ^−^ and TCRβ^+^ cells, which were prepared at 12 days after TCR stimulation. Combined data from three independent ChIP experiments is shown.

It has been shown that a subset of transcriptional machineries and chromatin-remodeling complexes are assembled at the Dβ1 promoter independently of Eβ function in T cell progenitors^19^. Another study showed that TCR stimulation induces changes in chromatin structure and gene expression at numerous genetic loci^20^. Therefore, we tested whether TCR stimulation could restore TCRβ expression from cells lacking Eβ, To this aim, we prepared splenic CD8^+^ T cells from *Eβ *^*f*/*f*^:*E8I*-*Cre* mice and activated them *in vitro* by antibody-mediated TCR stimulation. Interestingly, both TCRβ^−^ and TCRβ^+^ populations arose from CD8^+^ T cells of *Eβ *^*f*/*f*^:*E8I*-*Cre* mice over the course of activation, whereas the uniform and stable TCRβ expression was observed in control CD8^+^ T cells (Fig. 4A). CD8^+^ T cells from *Eβ *^*f*/*f*^:*E8I*-*Cre* mice showed a sign of delayed activation as shown with activation markers CD25 and CD69 one day after stimulation (Fig. 4B). The appearance of TCBβ^−^ cells after stimulation is not surprising since mature peripheral T cells already showed quite reduced levels of TCRβ as shown by *Eβ *^*f*/*f*^:*E8I*-*Cre* mice. In contrast to these cells, some proportion of cells after stimulation was able to maintain TCRβ expression in the absence of Eβ, suggesting that an Eβ-independent mechanism might operate after some points during prolonged cell proliferation triggered by TCR stimulation. TCRβ^+^ and TCRβ^−^ cells showed a similar rate of proliferation as shown in Fig. 4B. Comparison of histone modifications at the *Tcrb* locus between TCRβ^−^ and TCRβ^+^ cells by ChIP assay showed that H3K4me3, a known representative active epigenetic mark, was enriched throughout the *Tcrb* locus in TCRβ^+^ cells, while the *Tcrb* locus in TCRβ^−^ cells was mostly covered with H3K27me3, a representative mark for suppressive epigenetic modification (Fig. 4C). Correlation of epigenetic modifications with expression status of the *Tcrb* gene deficient for Eβ suggested that Eβ-independent mechanism, at least in part, compensates the Eβ function that retains active epigenetic modifications in activated T cells. Appearance of TCRβ^−^ cells with repressive epigenetic marks by loss of Eβ also suggests that Eβ might be necessary to maintain active epigenetic marks.

## Discussion

The *Tcrb* locus has been recognized as a useful model locus to understand the mechanisms of V(D)J recombination^21^, and the requirement of Eβ in the initiation of transcription and recombination of the DβJβ region has been well recognized^6^,^7^. However, it has remained unclear whether transcription and recombination are controlled by common or distinct DNA sequences within the Eβ. Similarly, Eβ function at later stages of thymocyte development has not been characterized mainly due to an arrest of T cell development at the DN stage by a lack of the Eβ. In this study, we showed that the single Runx site mutation had a distinct impact on recombination versus transcription in cells at late developmental stages. The loss of one Runx binding motif severely inhibited germline transcription at the Dβ segment and thus Dβ to Jβ recombination. A previous study showed that Runx complexes associate with pDβ promoters in an Εβ-dependent manner^22^. A different study also showed that Runx binding to the TCR Eδ enhancer precedes the binding of another binding proteins, c-Myb^23^. These observations suggested a possibility that Runx complex serves as scaffold proteins on Eβ enhancer to induce sequential binding of other *trans*-factors onto Eβ, thus acting as important mediators for holocomplexes formation between the pDβ promoters and the Eβ enhancer. Presumably, decreased affinity of Eβ with Runx complexes upon the loss of one docking site results in an unstable Runx binding, leading to a reduced probability of recruitment of sequential factors, including RAG-1/2^24^, onto the Dβ–Jβ segments. However, in a small proportion of DN thymocytes, *trans*-acting factor complexes that bridge the Eβ enhancer to pDβ promoters could be formed even with one Runx docking site for a certain amount of time sufficient to induce successful Dβ to Jβ recombination.

Whether the Eβ enhancer is involved in the activation of Vβ promoters remains to be clarified. Previous studies showed that the deletion of the Eβ enhancer had no significant effect on germline transcription, histone acetylation at the 5′ Vβ regions or long-range interaction of the Vβ segments with Dβ under germline configuration of the *Tcrb* locus^9^,^25^. However, our result showed that conditional deletion of Eβ by *Lck*-*Cre* led to a significant decrease of Vβ to DJβ joining. Furthermore, the removal of Eβ by *Cd4*-*Cre* after Vβ to DβJβ assembly quickly erased surface TCRβ expression from DP thymocytes. These results suggest that, after the relocation of Vβ to juxtapose Eβ, the activity of pVβ promoters becomes Eβ-dependent. Thus, our findings shed new light on Eβ function in the control of the activity of pVβ promoters. It is unclear how the dependency of pVβ promoters on Eβ is altered at a distinct developmental stage. Recent studies proposed the presence of a barrier element upstream of the Dβ1Jβ1 cluster with features of H3K4me3 accumulation^26^ and CTCF binding^25^, which would prevent pre-interaction of Vβ segments with the active DβJβ segment. It is possible that the removal of such a biological barrier by Vβ to DβJβ joining allows Eβ to communicate with Vβ promoter activity.

Because TCRβ expression on DP thymocytes was quickly lost upon Eβ removal, Eβ is likely to be a sole, or at least indispensable, enhancer to maintain *Tcrb* expression. Along with sustained TCRβ expression from the *Eβ*^*M1*^ and *Eβ*^*M2*^ allele in mature T cells as well as sustained TCRβ expression in T cells lacking Runx complex function^14^ (data not shown), this finding suggests that Eβ does not require Runx complexes at least after complete Vβ to Dβ assembly. This makes us wonder what the biological significance for Eβ to become independent of Runx complexes to maintain *Tcrb* expression from the rearranged *Tcrb* gene is. To secure mono antigen specificity on each T cell, once a functional TCRβ chain is produced, further recombination at the other allele is inhibited at the stage of the Vβ to DβJβ assembly, known as allelic exclusion. Although some models assuming distinct accessibilities^4^ have been proposed to explain how allelic exclusion is regulated, the precise molecular mechanisms remains unsolved. Irrespective of the mechanism, transcription on the included allele must be maintained while the Vβ to DβJβ rearrangement is inhibited on the excluded allele. Although the role of Eβ to facilitate Vβ to DβJβ assembly has not been described, this possibility is not formally discarded. Rather, our results of decreased Vβ to DβJβ joining by *Eβ* removal from DN cells by *Lck*-*Cre* suggested the involvement of Eβ in this reaction in addition to maintaining transcription from pVβ promoters. Given that Runx complexes are involved in holocomplex formation at Dβ to Jβ joining reaction^22^, it is possible that Runx complexes play a similar role in the formation of the second holocomplex at Vβ to DβJβ joining. If this is the case, the inhibition of Runx complex function on Eβ would result in the inhibition of Vβ to Dβ assembly, while transcription from rearranged *Tcrb* gene can be maintained. It is important to further clarify how Runx complex controls the Eβ activity and whether Eβ plays any role in controlling Vβ to Dβ rearrangement.

## Materials and Methods

### Mice

The 3.0 kb *BamHI*-*BamHI* fragment corresponding to the 5′ long arm of our targeting vector was cut from a plasmid containing genomic DNA from the *Tcrb* locus (a gift from Dr. Bories). The 3′ short arm and the Eβ region were PCR amplified from the same plasmid using primers containing the desired restriction enzyme sites at the both ends. For the construction of the *Eβ *^*flox*^ targeting vector, these fragments were ligated sequentially into the plasmid harboring a *thymidine kinase (TK*) gene and a *neomycin* resistance gene (*neo*^*r*^) cassette flanked by two loxP sequences. The M1, M2 and M3 mutation were created by an overlapping PCR, and were sequenced to confirm the mutations. Each mutant Eβ fragment containing an *HpaI* site at the 5′-end and a *SalI* site at the 3′-end was replaced with the wild-type Eβ fragment by ligation into HpaI/SalI digested targeting vector. Each targeting vector was linearized by *NotI* digestion, and was transfected into the E14 ES cells as described previously^27^. After homologous recombination was confirmed by Southern blotting, 20 μg of a pMC-Cre expression vector encoding Cre recombinase was transfected into each ES clone to remove the *neo*^*r*^ gene. Mutant mouse strains harboring the *Eβ*^*Δ*^, *Eβ *^*flox*^, *Eβ*^*M1*^, *Eβ*^*M2*^, or *Eβ*^*M3*^ mutation were established through germline transmission from chimera mice.

For the construction of the *E8I*-*Cre* transgene, a 1.9 kb LCR/TE (locus control region and thymocyte enhancer) region^28^ was PCR amplified from mouse genome DNA with primers to add an *XbaI* site at the 5′end, and was cloned into the pCR-TOPOII vector (Invitrogen). The 1.6 kb *HindIII*-*HindIII* fragment of the core *E8I* enhancer was excised together with the *Cd8a* promoter fragment from the plasmid^29^ by *EcoRV* and *XhoI* digestion. The 1.9 kb *XbaI*-*EcoRV* LCR/TE fragment and 2.1 kb *EcoRV*-*XhoI E8I*/*Cd8a* promoter fragment were ligated into the *XbaI* and *XhoI* digested pBluescript vector by trimolecular ligation, generating a pTE/E8I vector. The 6 kb *XhoI*-*XhoI* fragment containing the intronic region from the mouse *Cd4* locus followed by *Cre*-*ires*-*GFP* sequences was cut out from the previously described Cd*8*-*Cre* transgene plasmid^30^, and was cloned into the *XhoI* cleaved pTE/E8I vector. The *E8I*-*Cre* transgene was separated from the vector by *NotI* digestion, and was microinjected at J*apan SLC inc*. All mice were maintained in the animal facility at the RIKEN IMS, and all animal procedures were in accordance with protocol approved by the institutional guidelines for animal care.

### Flow cytometry analyses

All monoclonal antibodies used for cell staining were purchased from BD Biosciences. Intracellular staining was performed as previously described^31^. Stained cells were analyzed with a FACSCalibur (BD Biosciences) and data were analyzed using FlowJo software.

### RT-PCR and DNA-PCR

RT-PCR assays for germline transcription of the Dβ1 region were performed using total RNA from CD25^+^ DN cells purified using MACS (Miltenyi) microbeads. Cδ transcript was amplified by primers; 5′-agccagcctccggccaaaccatc-3′ and 5′-ctcttgggccatagcaaggctc-3′. DNA-PCR for analyzing Dβ to Jβ rearrangement and Vβ to DβJβ rearrangement were performed with 5000 sorted CD25^+^CD44^−^ DN3 thymocytes. Primers used for RNA-PCR and DNA-PCR were identical to those described previously^7^,^9^.

### T cell stimulation and culture

FACS sorted CD8^+^ T cells were stimulated with 2 μg/mL immobilized anti-CD3ε (553058: BD Biosciences) and 2 μg/mL soluble anti-CD28 antibody (553295: BD Biosciences) during the first two days. Cells were then maintained in the medium supplemented with 20 U/ml rIL-2 (11271164001: Roche) for additional days.

#### Chromatin Immunoprecipitation (ChIP) Assay

Chromatin-DNA was prepared from purified Lineage (B220, CD11b, CD11c, Gr-1 and Ter119)^−^CD3^−^CD4^−^CD8^−^TN thymocytes according to the protocol provided by the manufacturer of the ChIP assay kit (Upstate Biotechnology). Purity of cells after purification was at least over 98%. Rabbit anti-Runx1 serum used for Runx1-ChIP was previously described^32^. Control rabbit IgG (ab46540), anti-H3K4me3 (ab8580) and anti-H3K27me3 (ab6002) were from Abcam. DNA from input and immunoprecipitated chromatin DNA was subjected to PCR amplification. Primers to amply *Eβ*^*14*^, *Cd4 silencer (S4*)^33^ and other regions in the *Tcrb* locus^26^ for histone modifications were described previously.

## Additional Information

**How to cite this article:** Seo, W. *et al*. Distinct requirement of Runx complexes for TCRβ enhancer activation at distinct developmental stages. *Sci. Rep.* **7**, 41351; doi: 10.1038/srep41351 (2017).

**Publisher's note:** Springer Nature remains neutral with regard to jurisdictional claims in published maps and institutional affiliations.

## Supplementary Material

Supplementary Figures
