# Supplementary material for: Distinct requirement of Runx complexes for TCRβ enhancer activation at distinct developmental stages
Source: Sci Rep. 2017 Feb 2;7:41351. doi: 10.1038/srep41351 (PMC5288706; doi:10.1038/srep41351)
Supplement: Supplementary Figures [file srep41351-s1.pdf]

Distinct requirement of Runx complexes for TCRβ enhancer activation at distinct developmental stages

Wooseok Seo, Sawako Muroi, Kaori Akiyama and Ichiro Taniuchi

Laboratory for Transcriptional Regulation, RIKEN Center for Integrative Medical Sciences (IMS), 1-7-22 Suehiro-cho, Tsurumi-ku, Yokohama 230-0045, Japan.

Corresponding to Ichiro Taniuchi: ichiro.taniuchi@riken.jp

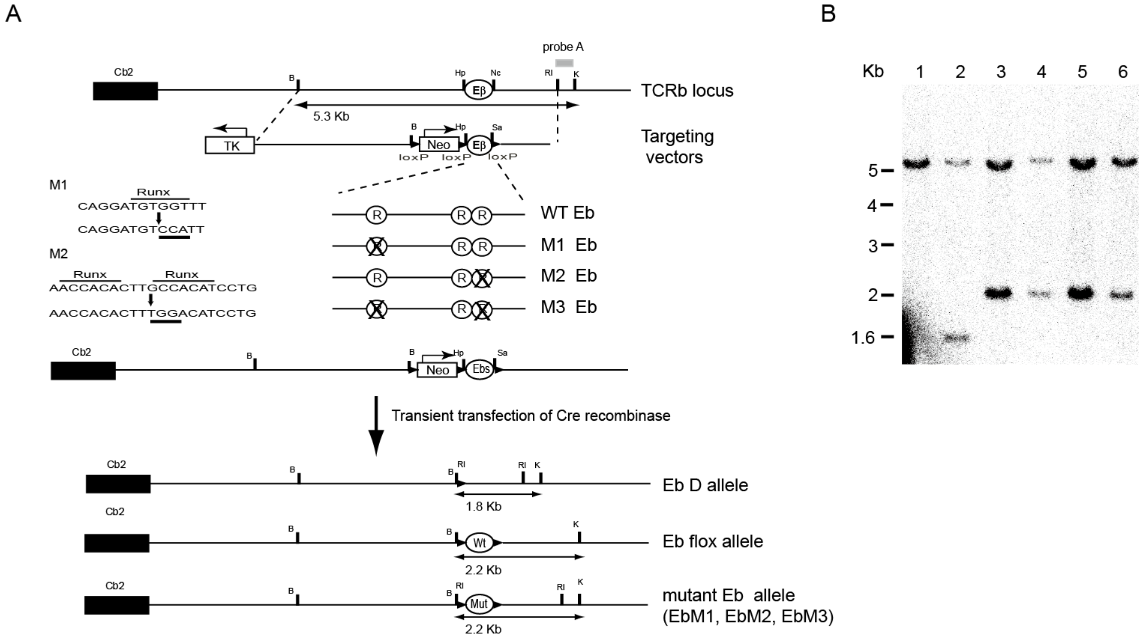

Supplementary figure 1. Strategies for targeting mutations into endogenous Eβ enhancer. A. Structure of murine *Tcrb* locus and targeting vectors. Black box, open circle, arrowhead represent the Cβ2 exon, the *Eβ* enhancer region and loxP sequences, respectively. Introduced M1 and M2 mutations, and Runx recognition sequences are marked by bold underline and thin upper line, respectively. The abbreviations for restriction enzymes are as follows B: *BamHI*, RI: *EcoRI*, K: *KpnI*, Hp: *HpaI*, Nc: *NcoI*. **B**. Southern blot analyses of each ES clone. After the removal of the neo^r gene by transient Cre expression, genomic DNA isolated from each ES clone was digested with BamHI and KpnI, and hybridized with probe shown in A as a grey box. Lanes 1, 2, 3, 3, 4, 5 and 6 are $E\beta^{+/+}$, $E\beta^{+/\Delta}$, $E\beta^{+/M1}$, $E\beta^{+/M2}$, $E\beta^{+/M3}$ and $Eb^{+/f}$ ES clones, respectively.

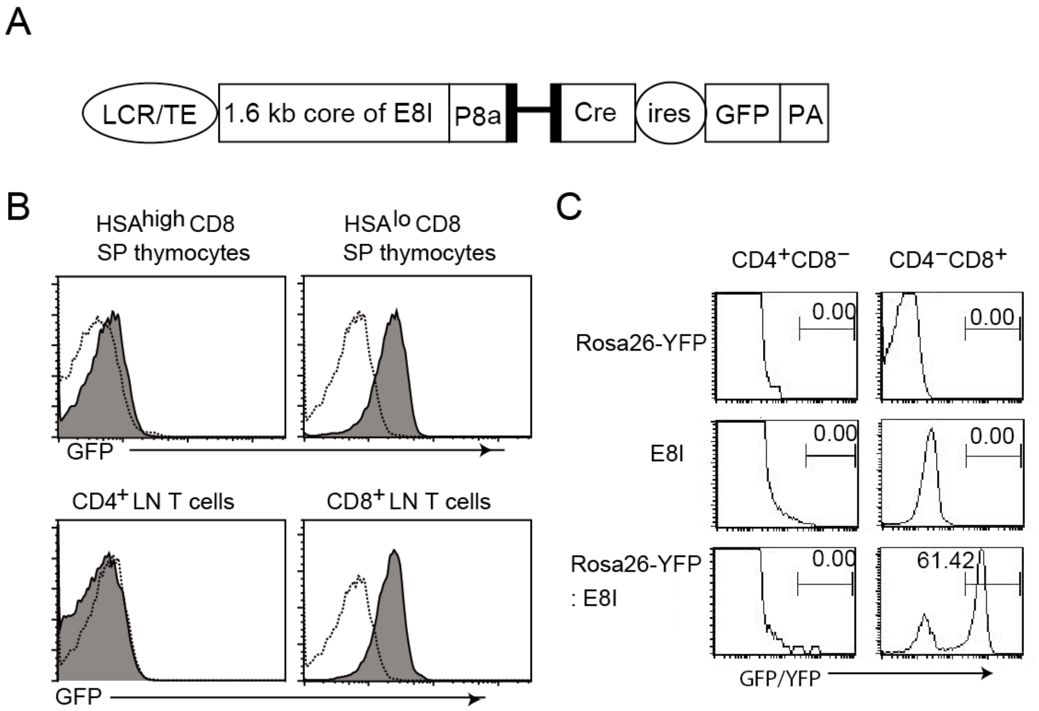

Supplementary figure 2. Generation of E8I-Cre transgenic mice for mature CD8-lineage specific Cre expression. **A.** Schematic structure of the *E8I-Cre* transgene. The transgene expression is driven by the combination of the 1.6 kb of core E8I enhancer and promoter of *Cd8α* gene (P8a). The black box and bold line represent a part of the exon and intron region, respectively, from the mouse *Cd4* locus. To monitor transgene expression, *ires-GFP* sequences were inserted at the downstream of the *Cre* gene. The LCR/TE region from the *ISOT* locus was inserted at the 5' end to avoid position effect variegation from the integrated site of the transgene. **B.** Expression of GFP during T cell development is shown as a histogram. The dotted line and shaded line represent GFP expression of cells from the non-transgenic control and *E8I-Cre* transgenic mouse, respectively. GFP expression was first detected in $CD4^{-}CD8^{+}$ SP thymocytes after HSA expression was downregulated. In peripheral T cells, GFP expression was detected specifically in $CD8^{+}$ T cells. **C**. Fate mapping approach showing the specificity and efficiency of *E8I-Cre* transgene mediated recombination at the Rosa26-STOP-YFP reporter locus. YFP expression from the Rosa26-STOP-YFP reporter allele was significantly higher than GFP expression from the *E8I-Cre* transgene, and was detected as a different peak in the histogram. Histograms showing GFP/YFP signals in lymph node $CD4^{+}$ and $CD8^{+}$ T cells from mice with the indicated genotype.
